# Lifetime exposure to brominated trihalomethanes in drinking water and swimming pool attendance are associated with chronic lymphocytic leukemia: a Multicase-Control Study in Spain (MCC-Spain)

**DOI:** 10.1038/s41370-023-00600-7

**Published:** 2023-09-19

**Authors:** Carolina Donat-Vargas, Manolis Kogevinas, Yolanda Benavente, Laura Costas, Elias Campo, Gemma Castaño-Vinyals, Guillermo Fernandez-Tardon, Javier Llorca, Inés Gómez-Acebo, Nuria Aragonés, Marina Pollan, Delphine Casabonne, Cristina M. Villanueva

**Affiliations:** 1 ISGlobal, Barcelona, Spain; 2 Universitat Pompeu Fabra (UPF), Barcelona, Spain; 3 CIBER Epidemiología y Salud Pública (CIBERESP), Madrid, Spain; 4 Unit of Cardiovascular and Nutritional Epidemiology, Intitute of Environmental Medicine, Karolinska Institutet, Stockholm, Sweden; 5 IMIM (Hospital del Mar Medical Research Institute), Barcelona, Spain; 6 Unit of Molecular and Genetic Epidemiology in Infections and Cancer (UNIC-Molecular), Cancer Epidemiology Research Programme, IDIBELL, Catalan Institute of Oncology, 08908 L’Hospitalet de Llobregat, Spain; 7 Haematopathology Section, Hospital Clınic of Barcelona, Institut d’Investigaciones Biomediques August Pi I Sunyer (IDIBAPS), University of Barcelona, Centrode Investigacion Biomedica en Red de Cancer (CIBERONC), Barcelona, Spain; 8 Health Research Institute of Asturias, ISPA, Oviedo, Spain; 9 Faculty of Medicine, University of Cantabria, Santander, Spain; 10 IDIVAL. Instituto de Investigación Sanitaria Valdecilla, 39011 Santander, Spain; 11 Epidemiology Section, Public Health Division, Department of Health of Madrid, Madrid, Spain; 12 Cancer and Environmental Epidemiology Unit, National Centre for Epidemiology, Carlos III Institute of Health, Madrid, Spain

**Keywords:** Water contaminants, Trihalomethanes, Nitrate, swimming pools, Cancer, Chronic lymphocytic leukemia

## Abstract

**Background:**

Chronic lymphocytic leukemia (CLL) etiology is poorly understood, and carcinogenic chemicals in drinking and recreational water are candidates.

**Objective:**

To evaluate the association between drinking-water exposure to trihalomethanes (THMs) and nitrate as well as lifetime swimming pool attendance and CLL.

**Methods:**

During 2010–2013, hospital-based CLL cases and population-based controls were recruited in Spain, providing information on residential histories, type of water consumed and swimming pool attendance. Average THMs and nitrate levels in drinking water were linked to lifetime water consumption. Odds ratios (OR) and 95% confidence intervals (CI) were estimated using mixed models.

**Results:**

Final samples for residential tap water analyses and swimming pool attendance analyses were 144 cases/1230 controls and 157 cases/1240 controls, respectively. Mean (SD) values for average lifetime residential brominated THMs and chloroform in tap water (μg/L), and ingested nitrate (mg/day) were 48.1 (35.6), 18.5 (6.7) and 13.7 (9.6) respectively in controls; and 72.9 (40.7), 17.9 (5.4), and 14.1 (8.8) in CLL cases. For each 10 μg/L increase of brominated THMs and chloroform lifetime-average levels, the ORs (95% CI) were 1.22 (1.14, 1.31) and 0.54 (0.34, 0.87), respectively. For each 5 mg/day increase of ingested nitrate, the OR of CLL was 0.91 (0.80, 1.04). The OR of lifetime pool users (vs. non-users) was 2.38 (1.61, 3.52). Upon performing annual frequency of attending pools analysis through categorization, the second and third categories showed an ORs of 2.36 (1.49, 3.72) and 2.40 (1.51, 3.83), respectively, and P-trend of 0.001.

**Impact statement:**

This study identifies an association of long-term exposure to THMs in drinking water, at concentrations below the regulatory thresholds and WHO guidelines, and swimming pool attendance, with chronic lymphocytic leukemia (CLL). These unprecedented findings are highly relevant since CLL is an incurable cancer with still unknown etiology and because the widespread exposure to chlorination by-products that remain in drinking and recreational water worldwide. Despite the demonstrated carcinogenicity in animals of several chlorination by-products, little is known about their potential risks on human health. This study makes a significant contribution to the search for environmental factors involved in the etiology of CLL and to the evidence of the health impact of these high prevalent water contaminants.

## Introduction

Chronic lymphocytic leukemia (CLL) is a hematologic neoplasm of memory B cells characterized by the clonal proliferation and accumulation of mature and typically CD5-positive B cells within the blood, bone marrow, lymph nodes, and/or spleen [1]. It is the most common type of leukemia in developed countries, with a current age-adjusted incidence of ~5 per 100 000 population [2]. CLL is more common in males than in females, in the elderly, with a mean age of diagnosis around 73 years, and, unlike other types of leukemia, CLL appears to be unique to persons of predominately European descent [3].

While enormous progress has been made in the management of CLL in the past decade to improve clinical outcomes, it is still an incurable cancer whose etiology is poorly understood. Family history of non-Hodgkin lymphoma or lymphoproliferative disorder, hepatitis C virus seropositivity, respiratory tract infections, history of atopic health disorders, reduced sun exposure, living on a farm or exposure to pesticides, and occupation as hairdresser are factors linked to CLL development [4, 5]. The elevated risk observed for occupational exposures of farming and hairdressers suggest that carcinogenic agents are likely involved in the pathogenesis of this blood disease. Thus, carcinogens contained both in drinking water and in recreational pools around the world such as some disinfection by-products (DBPs) and nitrate, are potential candidates.

DBPs constitute a complex mixture of chemicals formed as unintended by-products of the disinfectants applied to both drinking and recreational water to inactivate microbial pathogens [6]. Chlorine is a cost-effective disinfectant widely used worldwide and trihalomethanes are the most prevalent DBPs formed after chlorination. However, although disinfection is essential to avoid waterborne disease, it raises a public health issue: the potential carcinogenicity of DBPs [7]. Moreover, DBP concentrations in swimming pool water often exceed those found in drinking water. The primary routes of DBP uptake during swimming are dermal absorption and inhalation, that result in higher and more persistent levels of DBPs in the bloodstream compared to oral exposure, owing to their direct access to the bloodstream [8].

Nitrate occurrence in the water cycle is steadily increasing on a global scale, primarily due to the widespread use of nitrogen fertilizers and the intensification of agricultural practices [9]. Human exposure to nitrate mainly occurs through ingestion of food and drinking water [10]. When ingested, nitrate is reduced to nitrate, which can interact with endogenous molecules to form N-nitroso compounds such as nitrosamines and nitrosamides. Ingested nitrate leads to endogenous nitrosation to form nitrogenous compounds such as nitrosamines and nitrosamides, which are classified as probably carcinogenic to humans [11, 12]. Within this context and to provide more insight about the health impact of prevalent water contaminants, we explored the link between long-term exposures to THMs and nitrate in drinking water, as well as, the recreational use of swimming pools and CLL development.

## Methods

### Study design and population

CLL cases were recruited within the multicentric multicase-control (MCC) study in Spain (http://www.mccspain.org) in collaboration with the International Cancer Genome Consortium on Chronic Lymphocytic Leukemia Project (ICGC-CLL, www.cllgenome.es and www.icgc.org). Briefly, cases and controls were recruited between January 2010 and January 2013. CLL cases were identified in 11 hospitals from different Spanish regions, together with a set of frequency-matched controls by sex, age, and region. Controls were selected from the general population, identified from the lists of randomly selected family practitioners in primary health centers located within the catchment area of the hospitals recruiting CLL [13]. Response rates were 87% for cases and 53% for controls. Inclusion criteria required participants to be 20–85 years old, be able to understand and answer the questionnaire, and have lived for at least 6 months in the study area.

### Data collection

At recruitment, participants answered a structured computer-assisted questionnaire administered by trained personnel in a face-to-face interview to gather information on anthropometrics (self-reported), socio-demographics, lifestyle factors, and personal and family medical history. Cases and controls provided full address, year start and stop living in all the residences where they lived for at least 12 months since age 18 until the time of the interview, and the type of water consumed in each residence (municipal, bottled, well, other). Amount (glasses/day) of water ingested on average lifetime at home, work and other places was ascertained. Information related to swimming pool attendance included ever attendance (defined as more than 10 times in the lifetime), year (or age) when swimming pool attendance started and ended, average attendance frequency in summer and the rest of the year separately, average time in the water, and type of swimming pool (indoor, outdoor, both). Dietary habits the year before the interview were collected through a self-administered semiquantitative food frequency questionnaire, including a total of 140 food items, previously validated in Spain [14]. Questionnaires used are available online (http://mccspain.org). A final section evaluating the reliability of the interview was completed by the interviewer.

### Outcome definition

CLL cases were diagnosed according to the criteria of the International Workshop on Chronic Lymphocytic Leukemia [15]. CLL and small lymphocytic lymphoma are considered the same underlying disease. All diagnoses were morphologically and immunologically confirmed using flow cytometry immunophenotype and complete blood count. In the vast majority of CLL cases, we could differentiate between rai stage 0 or I–IV tumors. Given the generally indolent course of CLL, with most of the patients having a slow progression disease and with no treatment need, CLL cases were invited to participate independently of their date of diagnosis. Patients who received a CLL diagnosis within three years prior to the start of the study (January 2010) were eligible for inclusion. Incident cases were defined as patients diagnosed at during the study recruitment period (January 2010 – January 2013), while prevalent cases were defined as participants diagnosed ≤ 3 years prior to the start of the study (January 2010).

### THM and nitrate levels in municipal drinking water

We designed a structured questionnaire aimed at water utilities, local and/or health authorities to collect drinking water source (surface/ground water proportion) and treatment in the study areas back to 1940, as well as available THMs and nitrate measurements. We targeted data collection among study municipalities that contributed up to 80% of person-years. In addition, centralized routine monitoring data on THMs (chloroform, bromodichloromethane, dibromochloromethane and bromoform) and nitrate was provided by the SINAC (Spanish acronym of the National Information System on Water for Consumption) for the years 2004–2010. This data corresponded to samples collected at drinking water treatment plants and the distribution network, and included information at the water zone level fed by water supply operators from public or private companies or municipalities, and public or private laboratories. The water zone, that mostly corresponds to municipality, was defined as a geographical area supplied by water with a homogeneous source and treatment, and whose quality in the water distributed in the networks can be considered homogeneous. We linked each postal code from the residence to the corresponding water zone. The distribution of the sampling points and the sampling frequency varied greatly depending on the population served, extension of the water zone and the year, and could be more than once a day (e.g., Madrid), up to once every three months, or once a year in less populated areas. Measurements below the analytical limit of quantification (QL) were substituted with half the QL (QL/2) [16]. If the QL was missing, we imputed half of the most frequently reported QL.

### THM and nitrate levels in non-municipal drinking water

We measured nitrate in the 9 most-consumed bottled water brands in Spain using UV spectrophotometry, with 0.5/0.1 mg/L detection/quantification limit. Nitrate concentrations were in the range of 2.3–15.6 mg/l [17]. THMs were previously measured in 15 popular bottled water brands in Spain, through purge-and-trap and gas chromatography. Mean concentrations for chloroform and brominated THMs were ≤0.1 μg/L [18]; and limits of detection were 0.015 (chloroform), 0.004 (bromodichloromethane), 0.005 (dibromochloromethane) and 0.011 μg/L (bromoform). We used THM data from 56 measurements in different Spanish areas that were supplied by chlorinated ground water (e.g. wells). Average concentrations were 0.3, 0.3, 0.8, and 1.8 μg/L for chloroform, bromodichloromethane, dibromochloromethane, and bromoform, respectively. Nitrate data in private wells was not available.

### Estimation of long-term levels in municipal drinking water

We calculated the annual average level of nitrate and THMs at the water zone level. Years without measurements were assigned the average of all available measurements in the water zone if the water source and treatment did not change over the years. In the case of changes in the water source and/or treatment, procedures to back extrapolate were applied.

For THMs, since their concentrations in surface water are generally higher than in ground sources [19], we used surface water percentage as a weight to back extrapolate individual THM concentrations when water source changed through linear interpolation, assuming that concentrations increased proportionately to the percentage of surface water. Likewise, water zones with changes in treatment over the years and THM measurements were used to estimate the change percentage of THM concentrations after introducing such treatments. These percentages were applied as a weight to back-extrapolate THM concentrations in areas with changes in these specific treatments when measurements were unavailable. Before chlorination started, THMs concentrations were assumed to be zero. Total THMs (TTHM) levels were calculated by adding up chloroform, bromodichloromethane, dibromochloromethane, and bromoform concentrations.

For years without nitrate measurements in water zones where water source changed over the years, the ground water percentage was used as a weight to back-extrapolate concentrations using linear interpolation, assuming that nitrate levels increased with ground water proportion [9]. In municipalities without any nitrate measurement (covering ~0.5% of the total person-years), we imputed the levels of neighboring municipalities supplied with similar ground water proportion ± 10%.

### Individual indices of THM and nitrate exposure in the study population

#### Average THMs and nitrate levels in residential tap water

We used municipality and year to link levels in drinking water with residential histories of study participants from age 18 years to 2 years before the interview. We estimated the average concentration of nitrate (mg/L) and THMs (µg/L) for this period, henceforth referred to as “lifetime” or “long-term exposure”. In general, participants were assigned to different water zones based on their residence patterns. On average, individuals lived in three different residences during the exposure window period, with the residence at the time of the interview being the longest, spanning approximately 30 years.

#### Average ingested nitrate

To calculate waterborne ingested nitrate (mg/day), we assigned levels in drinking water by year according to the water type consumed at home, including municipal (tap), bottled, and private well/other water. Nitrate levels in municipal water were assigned for tap water consumption. Nitrate levels in the sampled bottled waters (range 2.3–15.6 mg/l) [17] were averaged using the sales frequency of each brand as a weight, leading to 6.1 mg/L of nitrate, that was assigned to study participants consuming bottled water. Since nitrate levels in well water were not available, waterborne ingested nitrate was considered missing for years when well water consumption was reported (~2%). The annual nitrate estimates were averaged from age 18 to 2 years before the interview and multiplied by the average daily water intake at the residence. Total amount of ingested water was ascertained as the number of water glasses per day consumed on average by the participant at home (L/day, assuming 200 mL/glass). Water intakes = 0 and above the 99th percentile (4 L/day), considered implausible, were treated as missing values in the analyses.

### Covariables

We collected information on self-reported age, education, weight and height 1 year before the interview to compute body mass index (BMI, kg/m^2^), family history of hematologic malignancy, smoking and physical activity. Smokers were defined as those smoking at least one cigarette/day for ≥6 months. Former smokers were defined as those who quit smoking ≥1 year before the interview. Physical activity was ascertained through open questions on any type of physical activity practiced in life, years, and frequency (hours/ week), to calculate metabolic equivalents (METs) from age 16 to 2 years before the interview.

### Statistical Analysis

**Fig. 1:**
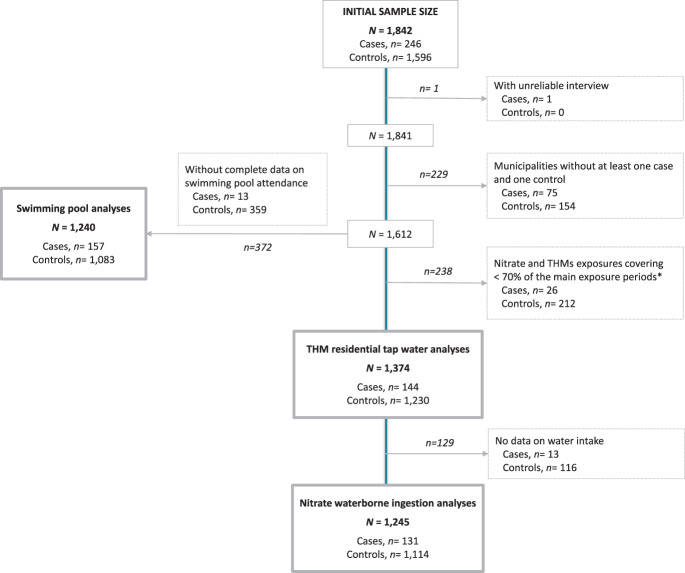
Flow chart showing exclusions of study participants from the Multicase-Control Study in Spain (MCC-Spain). Cases were defined as patients with CLL recruited within three years from diagnosis to interview (diagnosed ≤ 3 y prior to recruitment). * From 18 years of age to 2 years before the interview.

The initial sample consisted of 1,842 participants, comprising 246 CLL cases (92 incident, 154 prevalent) and 1,596 controls. Number of controls was greater than the number of cases because controls were selected to also be matched to other cancer sites. We excluded 1 subject with unreliable interview as qualified by the trained interviewers, and, in order to have a similar geographical distribution of cases and controls, only municipalities with at least one case and one control were included (*n* = 229 excluded). For the residential tap water analyses, participants with THMs or nitrate estimates covering less than 70% of the years between age 18 to 2 years before the interview were also excluded (*n* = 238), resulting in a sample of 1374 (144 cases, 1230 controls). For the nitrate waterborne ingestion analyses the sample was made up of 1245 (131 cases, 1114 controls) due to missing data on water consumption in 129 participants. Finally, out of 1612 there were 372 participants without complete data on swimming pool attendance and the sample for these analyses it was made up of 1240 (157 cases, 1803 controls) (Fig. 1).

As exposure to nitrate in drinking water is exclusively through the oral route, waterborne nitrate ingestion was evaluated as nitrate exposure. However, since inhalation and dermal absorption are suggested as relevant routes for THMs (volatile and permeable to the skin), residential tap water THM levels were used as main exposure. As a supplementary analysis, waterborne THM ingestion was additionally explored. Average lifetime residential tap water of total THMs, brominated THMs, chloroform (μg/L), and waterborne nitrate ingestion (mg/day), were treated as continuous and as tertiles defined according to the distribution among controls.

Our study also encompassed an evaluation of swimming pool attendance. Firstly, we conducted a comparative analysis between individuals classified as ‘users’ (defined as those who have attended the pool ≥10 times in their lifetime) and ‘non-users’ (defined as those who have attended the pool <10 times in their lifetime). Subsequently, we performed a secondary analysis by categorizing participants based on the average annual frequency of pool attendance. Given that over half of the participants fell into the non-user category, the reference category was designated as the group of ‘non-users, while users were further divided based on their attendance frequency, both below and above the median of the yearly average times of attendance. To achieve a finer-grained measurement, we further evaluated each successive 25th percentile increment of yearly pool attendance frequency, distinguishing between summer and rest of the year.

We estimated odds ratios (OR) and 95% confidence intervals (CI) of CLL using mixed models with recruitment area (Barcelona, Asturias, Cantabria) as random effect. To test for linear trends (P-trend) across increasing categories of exposure, the median concentration within each category was treated as a continuous variable in the model. Smoothed spline with three degrees of freedom from general additive models (GAM) were used to visually display the exposure–response relationships on continuous variables.

The selection of adjustment variables was made based on prior knowledge about CLL risk or preventive factors and variables potentially correlated with exposures of interest. Thus, all models were adjusted for recruitment area (introduced as random effect in the mixed model), age (<55, 55–64, 65–69, 70–74, ≥75 y) sex, and education (primary school or lower, secondary school, university). Further adjustment included family history of hematologic malignancy (yes/no), BMI (kg/m^2^), smoking (never, former, current), physical activity (inactive, low, moderate, very active), ever worked in farming or agriculture (yes/no) and alcohol consumption (never, former, current moderate consumption [(≤20 g/day men; ≤10 g/day women)], current high consumption [>20 g/day men; >10 g/day women]). An additional model was reported with mutual adjustments between nitrate waterborne ingestion, residential tap water chloroform and brominated THMs levels. Concerning the analysis of swimming pool attendance, the second model was not adjusted for physical activity, while the subsequent third model did include adjustments for physical activity. We used stochastic regression (which adds a random error term that appropriately reproduces the correlation between X and Y) to impute missing values in BMI (4%), physical activity (<1%) and alcohol consumption (18%). Treating the missing of the alcohol variable as a separate category had no impact on any estimate.

We explored the stratification by sex and by rai stage (0 and I–IV) and some sensitivity analyzes were further conducted: (i) excluding participants with family history of hematologic malignancy, to completely avoid any genetic influence and (ii) only including incident cases (i.e., CLL diagnosed after recruitment), to minimize the possibility of reverse causation. For this supplementary analysis, exposures were exclusively investigated in their continuous form, as this approach was necessitated by a considerable reduction in the sample size. All p values presented are two-tailed; <0.05 was considered statistically significant. Analyses were performed using STATA version 16.0 (Stata Corp, College Station, TX).

## Results

The recruitment area contributing with the largest population was Barcelona. The average age was 64 (11) years old for controls and 66 (10) for CLL cases. On average, cases had more often family history of hematologic malignancy, were less often current smokers, less physically active, worked more often in farming or agriculture, and consumed less alcohol (Table 1). The excluded participants exhibited no discernible differences in comparison to the selected sample (Supplementary Table 1).

**Table 1:** Characteristics of the study population.

***N*** = 1612	**Controls** ***N*** = 1442	**Cases** ***N*** = 170
Recruitment area, *n* (%)		
Barcelona	984 (68.2)	137 (80.6)
Asturias	221 (15.3)	26 (15.3)
Cantabria	237 (16.4)	7 (4.12)
Age (years), mean (SD)	64.1 (11.2)	66.2 (10.4)
<55, *n* (%)	288 (20.0)	27 (15.9)
55–64, *n* (%)	349 (24.2)	36 (21.2)
65–69, *n* (%)	286 (19.8)	34 (20.0)
70–74, *n* (%)	236 (16.4)	36 (21.2)
≥75, *n* (%)	283 (19.6)	37 (21.8)
Females, *n* (%)	622 (43.1)	56 (33.0)
Educational level, *n* (%)		
Primary school or lower	764 (53.0)	101 (59.4)
Secondary school	420 (23.1)	40 (23.5)
University	258 (17.9)	29 (17.1)
Family history of hematologic malignancy, *n* (%)	174 (12.1)	27 (15.9)
Body Mass Index (kg/m^2^), mean (SD)	26.9 (4.2)	27.1 (3.8)
Smoking, *n* (%)		
Never	614 (42.6)	67 (39.4)
Former	555 (38.5)	74 (43.5)
Current smoker	273 (18.9)	29 (17.1)
Physical activity, *n* (%)		
Inactive	395 (27.4)	54 (31.8)
Low	555 (38.5)	71 (41.8)
Moderate	210 (14.6)	20 (11.8)
Very active	282(19.6)	25 (14.7)
Ever worked in farming or agriculture, *n* (%)	323 (22.4)	53 (31.2)
Alcohol consumption, *n* (%)		
Never	197 (13.7)	17 (10.0)
Former	95 (6.6)	12 (7.1)
Current moderate consumption (≤20 g/day men; ≤10 g/day women)	782 (54.2)	113 (66.5)
Current high consumption (>20 g/day men; >10 g/day women)	368 (25.5)	28 (16.5)
Rai stage, *n* (%)		
0	─	105 (61.8)
I–IV	─	61 (35.9)
Unknown		4 (2.35)
**Average lifetime residential tap water**, **mean (SD)** ***N*** = **1374**	**Controls** ***N*** = **1230**	**Cases** ***N*** = **144**
Nitrate (mg/L)	8.82 (3.7)	9.7 (3.6)
Total trihalomethanes (μg/L)	66.6 (37.8)	90.8 (42.1)
Brominated trihalomethanes (μg/L)	48.1 (35.3)	72.9 (40.7)
Chloroform (μg/L)	18.5 (6.7)	17.9 (5.4)
**Average lifetime waterborne ingestion**, **mean (SD)** ***N*** = **1245**	**Controls** ***N*** = **1114**	**Cases** ***N*** = **131**
Nitrate (mg/d)	13.7 (9.6)	14.1 (8.8)
Total trihalomethanes (μg/d)	46 (55.2)	54.1 (72.4)
Brominated trihalomethanes (μg/d)	32 (42.9)	42.5 (62.9)
Chloroform (μg/d)	14 (15.9)	11.6 (12)
**Swimming pool attendance** ***N*** = **1240**	**Controls** ***N*** = **1083**	**Case** ***N*** = **157**
Users (≥10 times in life), *n* (%)	425 (39.2)	89 (57.7)
Average annual frequency (among users) (times/year), mean (SD)	89.9 (83.5)	77.5 (69.9)
Average frequency during summer (among users) (times/year), mean (SD)	41.0 (32.1)	35.4 (28.8)
Average frequency the rest of the year (among users) (times/year), mean (SD)	110.1 (65.8)	112.1 (50.1)

Brominated THMs includes bromodichloromethane, dibromochloromethane, and bromoform.

Total THMs (TTHMs) includes chloroform, bromodichloromethane, dibromochloromethane, and bromoform.

Cases of chronic lymphocytic leukemia (CLL) and controls from the MCC-Spain (Barcelona, Asturias, Cantabria).

Mean (SD) values for average lifetime residential tap water concentrations of brominated THMs (μg/L) and chloroform (μg/L), and waterborne ingested nitrate (mg/day) were 48.1 (35.6), 18.5 (6.7) and 13.7 (9.6), respectively, in controls; and 72.9 (40.7), 17.9 (5.4) and 14.1 (8.8) in CLL cases. While 58% of the cases exhibited a history of pool attendance over their lifespan, 39% of the control group manifested such attendance. Among users, average annual frequencies were slightly higher in controls than in cases (Table 1). Pool attendance during summer was 31% indoors, 67% outdoors and 2% mixed, while during the rest of the year, 98.5% was indoors, and 1.5% was outdoors (data not in tables).

### Trihalomethanes in residential tap water and nitrate waterborne ingestion

**Fig. 2:**
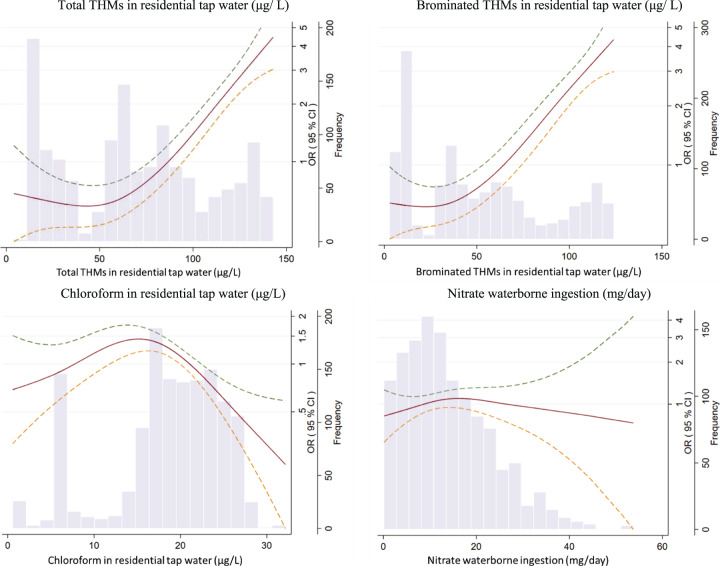
Exposure–response relationship of trihalomethanes in residential tap water, nitrate waterborne ingestion, and lifetime total swimming pool hours with chronic lymphocytic leukemia (CLL); expressed as odds ratios (ORs) and 95% confidence intervals (CIs). Smoothed spline with three degrees of freedom from general additive models adjusted for region, age, sex, and educational level, family history of hematologic malignancy, body mass index, smoking, physical activity, ever worked in farming or agriculture, alcohol consumption. The dashed lines represent the 95% confidence intervals (CIs).

The ORs of CLL were 1.24 (95%CI: 1.16, 1.34) and 1.22 (1.14, 1.31) for each 10 μg /L increase of, respectively, total and brominated THMs in residential tap water. Upon performing exposure analysis through tertile categorization, the third tertile showed an OR exceeding 2 for both total and brominated THMs. However, statistical significance was not attained. In contrast, an inverse association was identified between chloroform levels present in residential tap water and CLL, OR of 0.54 (0.34, 0.87) for 10 μg/L chloroform increase (Table 2, Fig. 2). These associations remained consistent and unchanged in both the stratified analyses and the sensitivity analyses (Table 3). However, the ingestion of waterborne THMs (total, brominated and chloroform) was not found to be associated with CLL (Supplementary Table 2).

**Table 2:** Association of trihalomethanes (THMs) in residential tap water and nitrate waterborne ingestion with chronic lymphocytic leukemia (CLL).

Exposure	Controls	Cases	OR (95% CI) Multivariable adjusted^a^	OR (95% CI) Multivariable adjusted^b^	OR (95% CI) Multivariable adjusted^c^
Total THMs in residential tap water (μg/L), *N* = 1374					
Tertile 1 (<52.2)	411	31	Ref. [1]	Ref. [1]	Ref. [1]
Tertile 2 (52.2–83.2)	409	23	0.48 (0.20, 1.19)	0.49 (0.19, 1.25)	0.57 (0.22, 1.48)
Tertile 3 (>83.2)	410	90	2.11 (0.91, 4.89)	2.13 (0.89, 5.14)	2.22 (0.90, 5.48)
P-trend	1230	144	<0.001	<0.001	<0.001
Per 10 μg /L	1230	144	1.24 (1.16, 1.32)	1.23 (1.15, 1.32)	1.24 (1.16, 1.34)
Brominated THMs in residential tap water (μg/ L), *N* = 1374					
Tertile 1 (<30.0)	411	31	Ref. [1]	Ref. [1]	Ref. [1]
Tertile 2 (30.0–60.8)	409	23	0.50 (0.21, 1.22)	0.52 (0.21, 1.29)	0.85 (0.31, 2.36)
Tertile 3 (>60.8)	410	90	2.16 (0.94, 4.94)	2.23 (0.95, 5.26)	2.32 (0.91, 5.91)
P-trend	1230	144	<0.001	<0.001	<0.001
Per 10 μg/L	1230	144	1.25 (1.17, 1.33)	1.24 (1.17, 1.3)	1.22 (1.14, 1.31)
Chloroform in residential tap water (μg/L), *N* = 1374					
Tertile 1 (<17.3)	410	54	Ref. [1]	Ref. [1]	Ref. [1]
Tertile 2 (17.3– 22.3)	410	62	0.69 (0.45, 1.06)	0.70 (0.45, 1.08)	0.81 (0.50, 1.31)
Tertile 3 (>22.3)	410	28	0.23 (0.13, 0.40)	0.23 (0.13, 0.41)	0.42 (0.22, 0.82)
P-trend	1230	144	<0.001	<0.001	0.02
Per 10 μg/L	1230	144	0.44 (0.32, 0.61)	0.44 (0.31, 0.61)	0.54 (0.34, 0.87)
Nitrate waterborne ingestion (mg/day), *N* = 1245					
Tertile 1 (<8.6)	372	34	Ref. [1]	Ref. [1]	Ref. [1]
Tertile 2 (8.6–16.8)	371	57	1.54 (0.91, 2.61)	1.57 (0.92, 2.68)	1.52 (0.87, 2.64)
Tertile 3 (>16.8)	371	40	0.94 (0.53, 1.66)	1.00 (0.56, 1.79)	1.04 (0.57, 1.89)
P-trend	1114	131	0.36	0.51	0.64
Per 5 mg/day	1114	131	0.93 (0.83, 1.04)	0.95 (0.84, 1.06)	0.91 (0.80, 1.04)

Odds ratios (OR) and 95% confidence intervals (CI) were calculated using mixed models with residential area (Barcelona, Asturias, Cantabria) as random effect.

Brominated THMs includes bromodichloromethane, dibromochloromethane, and bromoform.

Total THMs includes chloroform, bromodichloromethane, dibromochloromethane, and bromoform.

*ORs* Odds ratios, *95% CIs* 95% confidence intervals.

^a^Model adjusted for age, sex, and educational level.

^b^Model further adjusted for family history of hematologic malignancy, body mass index, smoking, physical activity, ever worked in farming or agriculture and alcohol consumption.

^c^Model further mutually adjusted for the other corresponding components, i.e., total THMs (nitrate model), nitrate (THMs model), chloroform and nitrate (brominated THMs model), brominated THMs and nitrate (chloroform model).

**Table 3:** Stratified analyses by chronic lymphocytic leukemia (CLL) rai stage and sex, and sensitivity analyses.

Stratification by:	CLL rai stage		Sex	
	Stage 0	Stage I-IV	Men	Women
Brominated THMs in residential tap water, OR^a^ (95% CI) Per 10 μg/L	1.21 (1.11, 1.31), (*N* = 1035)	1.23 (1.10, 1.37), (*N* = 1190)	1.26 (1.15, 1.39), (*N* = 736)	1.17 (1.04, 1.32), (*N* = 536)
Chloroform in residential tap water, OR^b^ (95% CI) Per 10 μg/L	0.57 (0.32, 1.03), (*N* = 1035)	0.38 (0.19, 0.77), (*N* = 1190)	0.46 (0.25, 0.85), (*N* = 736)	0.65 (0.31, 1.35), (*N* = 536)
Nitrate waterborne ingestion, OR^c^ (95% CI) Per 5 mg/day	0.95 (0.82, 1.11), (*N* = 1027)	0.87 (0.71, 1.07), (*N* = 1163)	0.93 (0.79, 1.10), (*N* = 724)	0.94 (0.76, 1.16), (*N* = 521)
Average frequency of pool attendance, OR^d^ (95% CI) Per 25 times/year increase^e^	1.06 (0.99, 1.14), (*N* = 1180)	1.00 (0.90, 1.11), (*N* = 1139)	1.07 (0.96, 1.18), (*N* = 521)	1.05 (0.97, 1.13), (*N* = 719)
**Excluding:**	**Family history of hematologic malignancy**	**Prevalent CLL cases**^**f**^		
Brominated THMs in residential tap water, OR^a^ (95% CI) Per 10 μg/L	1.22 (1.14, 1.32), (*N* = 1118)	1.21 (1.08, 1.37), (*N* = 1006)		
Chloroform in residential tap water, OR^b^ (95% CI) Per 10 μg/L	0.41 (0.25, 0.66), (*N* = 1118)	0.73 (0.31, 1.68), (*N* = 1006)		
Nitrate waterborne ingestion, OR^c^ (95% CI) Per 5 mg/day	0.91 (0.80, 1.05), (*N* = 1093)	0.76 (0.61, 0.95), (*N* = 998)		
Average frequency of pool attendance, OR^d^ (95% CI) Per 25 times/year increase^e^	1.06 (0.99, 1.13), (*N* = 1098)	1.05 (0.96, 1.16), (*N* = 914)		

Brominated THMs includes bromodichloromethane, dibromochloromethane, and bromoform.

*ORs* Odds ratios, *95% CIs* 95% confidence intervals.

^a^Model adjusted for region, age, sex, and educational level, family history of hematologic malignancy, body mass index, smoking, physical activity, ever worked in farming or agriculture, alcohol consumption, chloroform residential tap water and nitrate waterborne ingestion.

^b^Model adjusted for region, age, sex, and educational level, family history of hematologic malignancy, body mass index, smoking, physical activity, ever worked in farming or agriculture, alcohol consumption, brominated THM residential tap water and nitrate waterborne ingestion.

^c^Model adjusted for region, age, sex, and educational level, family history of hematologic malignancy, body mass index, smoking, physical activity, ever worked in farming or agriculture, alcohol consumption, total THM residential tap water.

^d^Model adjusted for region, age, sex, and educational level, family history of hematologic malignancy, body mass index, smoking, physical activity, ever worked in farming or agriculture and alcohol consumption.

^e^Corresponds to the 25th percentile among users.

^f^Only including newly diagnosed patients during the study period.

Waterborne ingested nitrate was consistently not associated with CLL. The OR associated for each 5 mg/day increase was 0.91 (0.80, 1.04), and when comparing tertile 3 vs. 1, the OR was 1.04 (0.57, 1.89; P-trend 0.64) (Table 2 and Fig. 2). Null associations for nitrate exposure remained in stratified and sensitivity analyses (Table 3).

### Swimming pool attendance

A positive association was observed between ever swimming pool attendance and CLL. The OR of swimming pool users (vs. non-users) was 2.38 (1.61, 3.52). Upon performing annual frequency of attending pools analysis through categorization, the second and third categories showed an ORs of 2.36 (1.49, 3.72) and 2.40 (1.51, 3.83), respectively (P-trend 0.001). For each 25-fold increment in the annual frequency of attending pools, the odds of CLL increased by 5%, OR = 1.05 (0.99, 1.12) (Table 4). This association was sustained both in attendance during the summer and winter months and remained similar in stratified and sensitivity analyses (Table 3).

**Table 4:** Association of average frequency of pool attendance with chronic lymphocytic leukemia (CLL).

Exposure	Controls	Cases	OR (95% CI) Multivariable adjusted^a^	OR (95% CI) Multivariable adjusted^b^	OR (95% CI) Multivariable adjusted^c^
Lifetime swimming pool attendance					
Non-users (<10 times in life)	658	65	Ref. [1]	Ref. [1]	Ref. [1]
Users (≥10 times in life)	425	89	2.08 (1.43, 3.03)	2.23 (1.52–3.27)	2.38 (1.61–3.52)
Average annual frequency					
Tertile 1 (non-users)	658	68	Ref. [1]	Ref. [1]	Ref. [1]
Tertile 2 (1 to <78 times/year)	228	47	2.07 (1.33, 3.23)	2.23 (1.42, 3.51)	2.36 (1.49, 3.72)
Tertile 3 (>78 times/year)	197	42	2.09 (1.34, 3.26)	2.23 (1.42, 3.50)	2.40 (1.51, 3.83)
P-trend	1083	157	0.004	0.002	0.001
Per 25 times/year increase^d^	1083	157	1.04 (0.98, 1.10)	1.04 (0.98, 1.11)	1.05 (0.99, 1.12)
Per 15 times/year increase during summer^d^	1015	149	1.08 (0.98, 1.18)	1.08 (0.98, 1.19)	1.09 (1.00, 1.20)
Per 40 times/year increase during the rest of the year^d^	872	104	1.14 (1.00, 1.30)	1.16 (1.02, 1.32)	1.19 (1.04, 1.36)

Odds ratios (OR) and 95% confidence intervals (CI) were calculated using mixed models with residential area (Barcelona, Asturias, Cantabria) as random effect.

Odds ratios (ORs) and 95% confidence intervals (CIs) *N* = 1240.

^a^Model adjusted for age, sex, and educational level.

^b^Model further adjusted for family history of hematologic malignancy, body mass index, smoking, ever worked in farming or agriculture and alcohol consumption.

^c^Model further adjusted for physical activity.

^d^Corresponds to the 25th percentile among users.

## Discussion

Despite the extensive use of chlorination as a cost-effective water disinfection method that has been applied for decades, there is surprising little evidence about its potential risks on human health. To our knowledge, this is the first study to evaluate the relationship between exposure to water contaminants, specifically THMs and nitrate in drinking water and swimming pool attendance, with CLL risk.

Elevated long-term total and brominated THM levels in residential tap water exhibited a positive association with an increased likelihood of developing CLL. Conversely, chloroform demonstrated an inverse association with CLL and no significant association was observed between nitrate and CLL. The absence of an association between THMs waterborne ingestion and CLL, in contrast to the observed association with residential THM levels, which account for different exposure routes, may suggest that inhalation and/or dermal exposures may play a more significant role in elevating the risk of CLL than THM ingestion. Swimming pool attendance was also associated with increased risk of CLL.

Chloroform, bromodichloromethane, dichloroacetic acid, and trichloroacetic acid have been classified as possible human carcinogens (Group 2B) by the International Agency for Research on Cancer [7, 20]. However, evidence of DBP carcinogenicity in animals differs by chemical, animal species, organ, and administration route. Since some DBPs (e.g., THMs) are volatile and skin permeable, exposure occurs through dermal absorption and inhalation when showering, bathing, hand dishwashing, cooking or attending swimming pools [21], in addition to ingestion. Long-term exposure to THMs, as a marker of DBP exposure, has been consistently associated with increased bladder cancer risk in epidemiological studies [22]. DPB exposure has also been linked to colorectal cancer, miscarriage, and birth defects, although evidence is less consistent [23, 24]. Exposure to swimming pool waters has also been related to eye and skin irritation, asthma and respiratory disorders [25, 26].

Drinking water chlorination form chloroform but also brominated THMs in the presence of bromide. In this study, mean levels of brominated THMs in drinking water were twice the levels of chloroform. Our data suggests that exposure to brominated THMs, but not chloroform, increases the risk of CLL, which could be expected given their different mechanisms of action and toxicological potential [26, 27]. Brominated THMs are more cytotoxic than chloroform in addition to being also genotoxic and mutagenic when tested under a wide range of experimental conditions (including human cells) [28, 29]. On the other hand, some animal data have observed that chloroform may have an inhibitory effect on certain tumors [30, 31]. In our sample, chloroform levels showed limited variability in the range where was most of the population. Associations should be cautiously interpreted and remain to be confirmed.

Among the 700 DBP identified, THMs and haloacetic acids are the most prevalent chlorination by-products, and have consequently been used in epidemiological studies as surrogates of total DBPs. However, there are other DBPs more toxic [32] such as haloacetonitriles, haloacetamides, or halonitromethanes [28, 33, 34]. Thus, a possible synergism or additive effect among the different DBPs in drinking water can be suspected and future studies should evaluate more completely the potential effects of combined exposures.

Our results on swimming pool attendance suggest that DBP exposure over the years in swimmers –which occurs through inhalation and dermal absorption, leading to a higher dose and longer duration in the bloodstream relative to oral exposure [8, 35, 36]—might be linked to CLL risk. Swimming pool water frequently exceeds DBP content relative drinking water due to the constant input of organic matter from swimmers and application of disinfectant [37]. The body fluids of the swimmers (such as urine, sweat, hair, skin cells) and personal care products (such as sunscreen and body lotions) together with other contaminants from leaves, dust or rainwater in the case of outdoor pools, react with the disinfectants used to prevent microbial proliferation –mostly chlorine, and to a lesser extend bromine or ozone– and forms unwanted DBPs. Hundreds of DBPs have also been identified in pools, including brominated THMs [38]. The DBP formation in swimming pools depends on several factors such as number of people in the pool, water temperature, and the amount of total organic carbon [39]. DBPs are reported to be significantly higher in the outdoor and recreational pools than in the indoors [40]. Outdoor and recreational pools are generally smaller in size and depth, with a greater influx of users, many of whom are children, which translates into a higher organic load and, therefore, a higher concentration of DBPs [41]. We, however, did not observed a greater risk of CLL associated with attendance to pools during summer (31% reported to be indoors, 67% outdoors and 2% mixed) than during the rest of the year (98.5% was indoors, and 1.5% was outdoors).

In a previous experimental study, in which we collected blood, urine, and exhaled air samples from adult volunteers before and after they swam for 40 min, we observed genotoxicity of swimming pool water and in swimmers. This genotoxicity was derived mainly from brominated THM but not chloroform [42]. The importance of brominated DBPs in the mutagenicity of recreational waters has been highlighted by other studies [43, 44]. Likewise, the presence of nitrogen-containing organic matter from bathers leads to the formation of nitrogenated by-products such as chloramines (mochloramine,dichloramine, trichloramine), haloacetonitriles and carcinogenic nitrosamines, also important drivers of toxicity [45, 46].

The main strengths of our study include the relatively large sample size that allowed us to analyze exposures using an updated and rigorous classification of CLL, and the long-term exposure approach, from 18 years of age to 2 years before the study interview. We were able to collect detailed individual information and to statistically adjust for a number of potential confounding factors. Residual confounding cannot be ruled out. Finally, the multicentric nature of the study allowed a wide geographic variability of THM and nitrate levels in residential tap water.

The limited historical measurements (particularly before 1980) and assumptions to model historical concentrations could reduce the accuracy of exposure estimates. However, to minimize exposure measurement error, we only included subjects with known exposures for at least 70% of the exposure window. Inability to account for exposures outside home and use of domestic filtration systems may have introduced nondifferential misclassification in the waterborne ingested estimates. However, the reported amount of water consumed at work (mean ± SD: 0.2 ± 0.3 L/day) and other places (0.01 ± 0.05 L/day) was smaller than that consumed at home (1.2 ± 0.7 L/day), and minor bias is expected. As for the use of domestic filters, a reduction of THMs levels has been reported [47]. There were no statistics on the use of domestic filters in Spain for the study exposure window, but expert knowledge suggests that the use of household water filters was probably uncommon. Overall, the expected effect on the associations from exposure measurement error is attenuation towards the null [48], as has been shown for other residence-based exposures [49].

In order to minimize potential recall bias in assessing exposure to swimming pools, we focused solely on individual average frequency of pool attendance, distinguishing between summer and the rest of the year. Attempting to obtain more precise information, such as the average hours spent in the pool during in life, would likely be prone to reporting errors. However, despite our efforts, we acknowledge that some degree of recall bias and exposure misclassification may be present. Notwithstanding, it is plausible that any such biases would be nondifferential, thereby tending to bias the association towards a null.

Personal information was collected retrospectively after diagnosis, and differential recall between cases and controls may not be totally ruled out. However, the questionnaire was administered by trained personnel in a face-to-face interview, and there is not an obvious link between the water questions and CLL that could motivate different responses between cases and controls. Moreover, the interviewers rated the quality of the interview and unreliable or inconsistent interviews were excluded from our analyses.

Selection bias arising from control sampling might be of concern since response rates were moderate, especially among controls, partly explained by the population-based source as opposed to hospital-based cases. However, there were no differences in the main characteristics between cases and controls and the probability of participation can be assumed to be independent from the exposure, thus, nondifferential bias is expected, if any. It was not possible to perform analyses by recruitment area due to the limited within area variability, but we conducted mixed models with area as random effect, which adjusts for environmental factors with potential different geographic distribution. Nevertheless, since correlation of these factors with our main exposures is unlikely, the expected impact on the results is minimal. Finally, the inclusion of prevalent cases might be another cause for attention since patients who survived might have a different etiology than those who died rapidly after diagnosis or might have modified their lifestyle, presumably towards healthier-like habits. However, sensitivity analysis suggested that the use of prevalent cases might not have introduced selective survival bias or reverse causality.

Our findings suggest that long-term exposure to brominated THMs in drinking water, as well as swimming pool attendance may have a role in the CLL etiology. Our unprecedented findings are highly relevant since no previous study has explored these associations. However, this is a first piece of evidence that needs to be confirmed in other studies and the biologic basis for the associations observed remains to be elucidated.

### Supplementary information

Reporting Checklist

Supplementary Tables

## Data Availability

Additional data that may be of interest to readers are available from the corresponding author on reasonable request.

## References

[1] Swerdlow SH, Campo E, Pileri SA, Harris NL, Stein H, Siebert R (2016). The 2016 revision of the World Health Organization classification of lymphoid neoplasms. Blood.

[2] NIH. The Surveillance Epidemiology and End Results (SEER) Program of the National Cancer Institute Cancer fact sheets: chronic lymphocytic leukemia (CLL) https://seer.cancer.gov/statfacts/html/clyl.html (Accessed 13th September 2023).

[3] Yang SM, Li JY, Gale RP, Huang XJ (2015). The mystery of chronic lymphocytic leukemia (CLL): why is it absent in Asians and what does this tell us about etiology, pathogenesis and biology?. Blood Rev.

[4] Benavente Y, Casabonne D, Costas L, Robles C, Alonso E, de la Banda E (2018). Established and suggested exposures on CLL/SLL etiology: results from the CLL-MCC-Spain study. Cancer Epidemiol.

[5] Slager SL, Benavente Y, Blair A, Vermeulen R, Cerhan JR, Costantini AS (2014). Medical history, lifestyle, family history, and occupational risk factors for chronic lymphocytic leukemia/small lymphocytic lymphoma: the InterLymph Non-Hodgkin Lymphoma Subtypes Project. J Natl Cancer Inst Monogr.

[6] Richardson S, Thruston A, Caughran T, Chen P, Collette T, Schenck K (2000). Identification of new drinking water disinfection by-products from ozone, chlorine dioxide, chloramine, and chlorine. Water Air Soil Pollut.

[7] International Agency for Research on Cancer. IARC, monographs on the evaluation of carcinogenic risk to humans. In: Trichloroethylene, tetrachloroethylene, and some other chlorinated agents: Lyon, France: IARC; 2014. PMC4781308 26214861

[8] Zare Afifi M, Blatchley ER (2015). Seasonal dynamics of water and air chemistry in an indoor chlorinated swimming pool. Water Res.

[9] Ward MH, Jones RR, Brender JD, de Kok TM, Weyer PJ, Nolan BT (2018). Drinking water nitrate and human health: an updated review. Int J Environ Res Public Health.

[10] Espejo-Herrera N, Gracia-Lavedan E, Pollan M, Aragones N, Boldo E, Perez-Gomez B (2016). Ingested nitrate and breast cancer in the Spanish Multicase-Control Study on Cancer (MCC-Spain). Environ Health Perspect.

[11] Grosse Y, Baan R, Straif K, Secretan B, El Ghissassi F, Cogliano V (2006). Carcinogenicity of nitrate, nitrite, and cyanobacterial peptide toxins. Lancet Oncol.

[12] IARC Working Group on the Evaluation of Carcinogenic Risks to Humans. IARC monographs on the evaluation of carcinogenic risks to humans. Ingested nitrate and nitrite, and cyanobacterial peptide toxins. IARC Monogr Eval Carcinog Risks Hum. 2010;94:1–412. PMC4781178 21141240

[13] Castano-Vinyals G, Aragones N, Perez-Gomez B, Martin V, Llorca J, Moreno V (2015). Population-based multicase-control study in common tumors in Spain (MCC-Spain): rationale and study design. Gac Sanit.

[14] Martin-Moreno JM, Boyle P, Gorgojo L, Maisonneuve P, Fernandez-Rodriguez JC, Salvini S (1993). Development and validation of a food frequency questionnaire in Spain. Int J Epidemiol.

[15] Hallek M, Cheson BD, Catovsky D, Caligaris-Cappio F, Dighiero G, Dohner H (2008). Guidelines for the diagnosis and treatment of chronic lymphocytic leukemia: a report from the International Workshop on Chronic Lymphocytic Leukemia updating the National Cancer Institute-Working Group 1996 guidelines. Blood.

[16] Croghan C, Egeghy PP. Methods of dealing with values below the limit of detection using SAS. St. Petersburg, FL: Southern SAS User Group; 2003.

[17] Espejo-Herrera N, Kogevinas M, Castano-Vinyals G, Aragones N, Boldo E, Ardanaz E (2013). Nitrate and trace elements in municipal and bottled water in Spain. Gac Sanit.

[18] Font-Ribera L, Kogevinas M, Nieuwenhuijsen MJ, Grimalt JO, Villanueva CM (2010). Patterns of water use and exposure to trihalomethanes among children in Spain. Environ Res.

[19] Villanueva CM, Kogevinas M, Grimalt JO (2003). Haloacetic acids and trihalomethanes in finished drinking waters from heterogeneous sources. Water Res.

[20] International Agency for Research on Cancer, et al. IARC, Monographs on the Evaluation of Carcinogenic Risk to Humans. Some chemicals present in industrial and consumer products. Food and Drinking-Water Lyon (France). 2016;101:349–74. PMC4939416 24772663

[21] Villanueva CM, Cordier S, Font-Ribera L, Salas LA, Levallois P (2015). Overview of disinfection by-products and associated health effects. Curr Environ Health Rep.

[22] Costet N, Villanueva CM, Jaakkola JJ, Kogevinas M, Cantor KP, King WD (2011). Water disinfection by-products and bladder cancer: is there a European specificity? A pooled and meta-analysis of European case-control studies. Occup Environ Med.

[23] Save-Soderbergh M, Toljander J, Donat-Vargas C, Akesson A (2021). Drinking water disinfection by-products and congenital malformations: a nationwide register-based prospective study. Environ Health Perspect.

[24] Nieuwenhuijsen MJ, Martinez D, Grellier J, Bennett J, Best N, Iszatt N (2009). Chlorination disinfection by-products in drinking water and congenital anomalies: review and meta-analyses. Environ Health Perspect.

[25] Villanueva CM, Font-Ribera L (2012). Health impact of disinfection by-products in swimming pools. Ann Ist Super Sanita.

[26] Font-Ribera L, Marco E, Grimalt JO, Pastor S, Marcos R, Abramsson-Zetterberg L (2019). Exposure to disinfection by-products in swimming pools and biomarkers of genotoxicity and respiratory damage - the PISCINA2 Study. Environ Int.

[27] Cortes C, Marcos R (2018). Genotoxicity of disinfection byproducts and disinfected waters: a review of recent literature. Mutat Res Genet Toxicol Environ Mutagen.

[28] Richardson SD, Plewa MJ, Wagner ED, Schoeny R, Demarini DM (2007). Occurrence, genotoxicity, and carcinogenicity of regulated and emerging disinfection by-products in drinking water: a review and roadmap for research. Mutat Res.

[29] de Castro Medeiros L, de Alencar FLS, Navoni JA, de Araujo ALC, do Amaral VS (2019). Toxicological aspects of trihalomethanes: a systematic review. Environ Sci Pollut Res Int.

[30] Daniel FB, Reddy TV, Stober JA, Olson GR (1991). Site-specific modulation of carcinogen-induced gastrointestinal tract nuclear anomalies in B6C3F1 mice by chloroform. Anticancer Res.

[31] Zhang L, Cai Q, Lin J, Fang Y, Zhan Y, Shen A (2014). Chloroform fraction of Scutellaria barbata D. Don promotes apoptosis and suppresses proliferation in human colon cancer cells. Mol Med Rep.

[32] Plewa MJ, Muellner MG, Richardson SD, Fasano F, Buettner KM, Woo YT (2008). Occurrence, synthesis, and mammalian cell cytotoxicity and genotoxicity of haloacetamides: an emerging class of nitrogenous drinking water disinfection byproducts. Environ Sci Technol.

[33] Ding S, Chu W, Krasner SW, Yu Y, Fang C, Xu B (2018). The stability of chlorinated, brominated, and iodinated haloacetamides in drinking water. Water Res.

[34] Allen JM, Plewa MJ, Wagner ED, Wei X, Bokenkamp K, Hur K (2022). Drivers of disinfection byproduct cytotoxicity in U.S. DRinking Water: Should Other DBPs be considered for regulation?. Environ Sci Technol.

[35] Ashley DL, Blount BC, Singer PC, Depaz E, Wilkes C, Gordon S (2005). Changes in blood trihalomethane concentrations resulting from differences in water quality and water use activities. Arch Environ Occup Health.

[36] Leavens TL, Blount BC, DeMarini DM, Madden MC, Valentine JL, Case MW (2007). Disposition of bromodichloromethane in humans following oral and dermal exposure. Toxicol Sci.

[37] Carter RAA, Allard S, Croue JP, Joll CA (2019). Occurrence of disinfection by-products in swimming pools and the estimated resulting cytotoxicity. Sci Total Environ.

[38] Richardson SD, DeMarini DM, Kogevinas M, Fernandez P, Marco E, Lourencetti C (2010). What’s in the pool? A comprehensive identification of disinfection by-products and assessment of mutagenicity of chlorinated and brominated swimming pool water. Environ Health Perspect.

[39] Chu H, Nieuwenhuijsen MJ (2002). Distribution and determinants of trihalomethane concentrations in indoor swimming pools. Occup Environ Med.

[40] Font-Ribera L, Esplugues A, Ballester F, Martinez-Arguelles B, Tardon A, Freire C (2010). [Trihalomethanes in swimming pool water in four areas of Spain participating in the INMA project]. Gac Sanit.

[41] Carter RAA, Joll CA (2017). Occurrence and formation of disinfection by-products in the swimming pool environment: A critical review. J Environ Sci.

[42] Kogevinas M, Villanueva CM, Font-Ribera L, Liviac D, Bustamante M, Espinoza F (2010). Genotoxic effects in swimmers exposed to disinfection by-products in indoor swimming pools. Environ Health Perspect.

[43] Weaver WA, Li J, Wen Y, Johnston J, Blatchley MR, Blatchley ER (2009). Volatile disinfection by-product analysis from chlorinated indoor swimming pools. Water Res.

[44] Liberatore HK, Daiber EJ, Ravuri SA, Schmid JE, Richardson SD, DeMarini DM (2022). Disinfection byproducts in chlorinated or brominated swimming pools and spas: role of brominated DBPs and association with mutagenicity. J Environ Sci.

[45] Jurado-Sanchez B, Ballesteros E, Gallego M (2010). Screening of N-nitrosamines in tap and swimming pool waters using fast gas chromatography. J Sep Sci.

[46] Walse SS, Mitch WA (2008). Nitrosamine carcinogens also swim in chlorinated pools. Environ Sci Technol.

[47] Egorov AI, Tereschenko AA, Altshul LM, Vartiainen T, Samsonov D, LaBrecque B (2003). Exposures to drinking water chlorination by-products in a Russian city. Int J Hyg Environ Health.

[48] Rothman KJ, Greenland S, Lash TL. Modern epidemiology. Vol. 3. Philadelphia: Wolters Kluwer Health/Lippincott Williams & Wilkins; 2008.

[49] Wei Y, Qiu X, Yazdi MD, Shtein A, Shi L, Yang J (2022). The impact of exposure measurement error on the estimated concentration-response relationship between long-term exposure to PM2.5 and mortality. Environ Health Perspect.

