# Supplementary material for: Lifetime exposure to brominated trihalomethanes in drinking water and swimming pool attendance are associated with chronic lymphocytic leukemia: a Multicase-Control Study in Spain (MCC-Spain)
Source: J Expo Sci Environ Epidemiol. 2023 Sep 19;34(1):47–57. doi: 10.1038/s41370-023-00600-7 (PMC10907291; doi:10.1038/s41370-023-00600-7)
Supplement: Supplementary file 2 — Supplementary Tables [file 41370_2023_600_MOESM2_ESM.docx]

| **Table S1**. Characteristics of cases of chronic lymphocytic leukaemia (CLL) and controls from the MCC-Spain study excluded from the analysis*. ***N* = 597** | | |
| --- | --- | --- |
|  | **Controls**  **N=482** | **Cases**  **N=115** |
| Recruitment area, *n* (%) |  |  |
| Barcelona | 207 (43.0) | 102 (88.7) |
| Asturias | 66 (13.7) | 10 (8.70) |
| Cantabria | 209 (43.4) | 3 (2.61) |
| Age (years), mean (SD) |  |  |
| <55, *n* (%) | 154 (32.0) | 18 (15.7) |
| 55-64, *n* (%) | 107 (22.2) | 37 (32.2) |
| 65-69, *n* (%) | 80 (16.6) | 24 (20.9) |
| 70-74, *n* (%) | 51 (10.6) | 12 (10.4) |
| ≥75, *n* (%) | 90 (18.7) | 24 (20.9) |
| Females, *n* (%) | 223 (46.3) | 42 (36.5) |
| Educational level, *n* (%) |  |  |
| Primary school or lower | 263 (54.6) | 66 (57.4) |
| Secondary school | 137 (28.4) | 26 (22.6) |
| University | 82 (17.0) | 23 (20.0) |
| Family history of hematologic malignancy, *n* (%) | 56 (11.6) | 21 (18.3) |
| Body Mass Index (kg/m^2^), mean (SD) | 26.9 (4.6) | 27.0 (3.9) |
| Smoking, *n* (%) |  |  |
| Never | 229 (47.5) | 53 (46.1) |
| Former | 167 (34.7) | 43 (37.4) |
| Current smoker | 86 (17.8) | 19 (16.5) |
| Physical activity, *n* (%) |  |  |
| Inactive | 161 (33.4) | 27 (23.5) |
| Low | 141 (29.3) | 46 (40.0) |
| Moderate | 74 (15.4) | 14 (12.2) |
| Very active | 106 (22.0) | 28 (23.4) |
| Ever worked in farming or agriculture, *n* (%) | 129 (26.8) | 40 (34.8) |
| Alcohol consumption, *n* (%) |  |  |
| Never | 86 (17.8) | 11 (9.6) |
| Former | 36 (7.47) | 13 (11.3) |
| Current moderate consumption (≤ 20 g/day men; ≤ 10 g/day women) | 257 (53.3) | 76 (66.1) |
| Current high consumption (> 20 g/day men; > 10 g/day women) | 103 (21.4) | 15 (13.0) |
| Rai stage, *n* (%) |  |  |
| 0 | ─ | 73 (63.5) |
| I-IV | ─ | 39 (33.9) |
| Unknown |  | 3 (2.61) |
| **Average lifetime residential tap water**, mean (SD) |  |  |
| Nitrate (mg/L) | 8.1 (3.4) | 10.1 (3.5) |
| Total trihalomethanes (μg/L) | 50.4 (36.9) | 89.2 (42.6) |
| Brominated trihalomethanes (μg/L) | 35.6 (32.9) | 72.9 (40.6) |
| Chloroform (μg/L) | 14.8 (8.2) | 16.3 (7.7) |
| **Average lifetime** **waterborne ingestion**, mean (SD) |  |  |
| Nitrate (mg/d) | 10.1 (8.1) | 13.4 (8.4) |
| Total trihalomethanes (μg/d) | 33.8 (56.0) | 45.4 (78.0) |
| Brominated trihalomethanes (μg/d) | 22.2 (40.9) | 37.4 (67.4) |
| Chloroform (μg/d) | 11.65 (18.6) | 7.97 (12.1) |
| * Participants excluded because their interview was unreliable, or were from municipalities without at least one case and one control, or because nitrate and THMs exposures covered < 70% of the total exposure period or because no reported data on water intake (n=597 out of 1842).  Brominated THMs includes bromodichloromethane, dibromochloromethane, and bromoform. Total THMs (TTHMs) includes chloroform, bromodichloromethane, dibromochloromethane, and bromoform. | | |

| **Table S2.** Association of trihalomethanes (THMs) waterborne ingestion with chronic lymphocytic leukaemia (CLL). Odds ratios (ORs) and 95% confidence intervals (CIs). | | | | | |
| --- | --- | --- | --- | --- | --- |
| **Exposure** | **Controls** | **Cases** | **OR (95% CI)**  **Multivariable adjusted^1^** | **OR (95% CI)**  **Multivariable adjusted^2^** | **OR (95% CI)**  **Multivariable adjusted^3^** |
| **Total** **THMs waterborne ingestion (μg/day),** N=1,244 | |  |  |  |  |
| Tertile 1 (<10.8) | 371 | 39 | Ref. 1 | Ref. 1 | Ref. 1 |
| Tertile 2 (10.8-46.3) | 371 | 43 | 1.09 (0.68, 1.73) | 1.10 (0.69, 1.75) | 1.06 (0.66, 1.70) |
| Tertile 3 (>46.3) | 371 | 49 | 1.01 (0.63, 1.60) | 1.02 (0.64, 1.63) | 1.11 (0.66, 186) |
| P-trend | 1,113 | 131 | 0.93 | 0.98 | 0.71 |
| Per 10 μg /day | 1,113 | 131 | 1.01 (0.98-1.05) | 1.01 (0.98-1.05) | 1.04 (0.99-1.08) |
| **Brominated THMs waterborne ingestion (****μg/ day),** N=1,244 | |  |  |  |  |
| Tertile 1 (<5.9) | 371 | 43 | Ref. 1 | Ref. 1 | Ref. 1 |
| Tertile 2 (5.9-29.2) | 371 | 37 | 0.85 (0.53, 1.36) | 0.86 (0.54, 1.38) | 0.69 (0.35, 1.36) |
| Tertile 3 (>29.2) | 371 | 51 | 0.95 (0.60, 1.51) | 0.97 (0.61, 1.54) | 0.84 (0.35, 2.02) |
| P-trend | 1,113 | 131 | 0.99 | 0.95 | 0.66 |
| Per 10 μg/day | 1,113 | 131 | 1.03 (0.99, 1.08) | 1.03 (0.99, 1.08) | 1.14 (1.07, 1.22) |
| **Chloroform waterborne ingestion (μg/day),** N=1,244 | |  |  |  |  |
| Tertile 1 (<3.7) | 371 | 41 | Ref. 1 | Ref. 1 | Ref. 1 |
| Tertile 2 (3.7-14.9) | 371 | 47 | 1.20 (0.76, 1.88) | 1.22 (0.77, 1.91) | 1.47 (0.74, 2.94) |
| Tertile 3 (>14.9) | 371 | 43 | 0.92 (0.58, 1.46) | 0.93 (0.58, 1.47) | 1.19 (0.52, 2.76) |
| P-trend | 1,113 | 131 | 0.59 | 0.60 | 1.00 |
| Per 10 μg/day | 1,113 | 131 | 0.87 (0.75, 1.02) | 0.88 (0.75, 1.02) | 0.66 (0.52, 0.84) |
| **Odds ratios (OR) and 95% confidence intervals (CI) were calculated using mixed models with residential area (Barcelona, Asturias, Cantabria) as random effect.**  Brominated THMs includes bromodichloromethane, dibromochloromethane, and bromoform  Total THMs includes chloroform, bromodichloromethane, dibromochloromethane, and bromoform.  **1** Model adjusted for age, sex, and educational level  **2** Model further adjusted for family history of hematologic malignancy, body mass index, smoking, physical activity, ever worked in farming or agriculture and alcohol consumption.  **3** Model further mutually adjusted for the other corresponding components, i.e., nitrate (THMs model), chloroform and nitrate (brominated THMs model), brominated THMs and nitrate (chloroform model). | | | | | |
